# The Phosphate Transporter Gene *OsPht1;4* Is Involved in Phosphate Homeostasis in Rice

**DOI:** 10.1371/journal.pone.0126186

**Published:** 2015-05-13

**Authors:** Ying Ye, Jing Yuan, Xiaojian Chang, Meng Yang, Lejing Zhang, Kai Lu, Xingming Lian

**Affiliations:** National Key Laboratory of Crop Genetic Improvement and National Center of Plant Gene Research (Wuhan), Huazhong Agricultural University, Wuhan, 430070, P.R. China; INRA, FRANCE

## Abstract

A total of 13 phosphate transporters in rice (*Oryza sative*) have been identified as belonging to the *Pht1* family, which mediates inorganic phosphate (Pi) uptake and transport. We report the biological property and physiological role of *OsPht1;4* (*OsPT4*). Overexpressing *OsPT4* resulted in significant higher Pi accumulation in roots, straw and brown rice, and suppression of *OsPT4* caused decreased Pi concentration in straw and brown rice. Expression of the *β*-glucuronidase reporter gene driven by the *OsPT4* promoter showed that *OsPT4* is expressed in roots, leaves, ligules, stamens, and caryopses under sufficient Pi conditions, consistent with the expression profile showing that *OsPT4* has high expression in roots and flag leaves. The transcript level of *OsPT4* increased significantly both in shoots and roots with a long time Pi starvation. *OsPT4* encoded a plasma membrane—localized protein and was able to complement the function of the Pi transporter gene PHO84 in yeast. We concluded that *OsPT4* is a functional Pi-influx transporter involved in Pi absorption in rice that might play a role in Pi translocation. This study will enrich our understanding about the physiological function of rice *Pht1* family genes.

## Introduction

Phosphorous is a major macronutrient for plant growth and development [1]. Inorganic phosphorus (Pi) ensures the maintenance of metabolism and the processes of cell formation and signal transduction [2–4]. To resolve the conflict between the root peripheral environment with low Pi concentrations (<10 μm) and high Pi concentrations (5–20 mM) in plant tissues [5], plants have formed an effective system to absorb and distribute Pi as much as possible. Two kinds of Pi transporters (PTs) have evolved to mediate Pi absorption and transport in rice: one is the high-affinity Pi transporter (with Km values in the micromolar concentration range) and the other is the low-affinity transporter (millinolar levels) [6–8]. After the first high-affinity Pi transporter PHO84 was found in yeast (*Saccharomyces cerevisiae*), many other Pi transporters, such as 19 genes in *Arabidopsis* and 26 genes in rice, have been identified [1,9]. Based on their difference in structure and subcellular localization, all Pi transporters from dicot and monocot plants have been grouped into four subfamilies, PHT1 to PHT4, which are mainly localized to plasma membrane, chloroplast, mitochondria, and Golgi apparatus, respectively [10].

In *Arabidopsis*, five of the nine genes in the *Pht1* family, which contains *AtPht1;1*, *AtPht1;4*, *AtPht1;5*, *AtPht1;8*, and *AtPht1;9*, have been isolated and functionally characterized [11,12]. In general, all five transporters belong to the high-affinity Pi transport system and are significantly affected by low Pi. *AtPht1;1* and *AtPht1;4* are capable of Pi transport and are localized mainly at the plasma membrane of epidermis and stele cells of *Arabidopsis* roots, confirming their important roles in Pi acquisition and root-to-shoot translocation [13]. *AtPht1;8* and *AtPht1;9* also show high expression levels in roots, suggesting their potential in root Pi uptake and translocation [14]. In contrast to those four genes, *AtPht1;5* is expressed in shoots and actively involved in the mobilization of phosphorous from the Pi source to sink organs in accordance with Pi status [15]. Aside from these transporters in *Arabidopsis*, many PT family genes from other species have also been characterized, such as PHO5 from *Neurospora crassa* [16], GvPT from *Glomus versiforme* [16,17], StPT1 and StPT2 from *potato* [18], LePT1 and LePT2 from *tomato* [19,20], MtPT1 and MtPT2 from *Medicago* [21], and HvPT1, HvPT2, and HvPT6 from *barley* [22–24]. All these genes have been shown to encode functional Pi transporter proteins and to be involved in Pi uptake or translocation.

So far, 13 genes have been isolated in the rice *Pht1* family and eight members have been functionally characterized [25]. Among them, *OsPT1*, *OsPT6*, *OsPT9*, and *OsPT10* are highly expressed in roots and responsible for absorbing Pi in rice [26–28]. *OsPT1* is constitutively expressed in roots, indicating that this gene plays an important role in Pi acquisition under various Pi conditions [26]. In contrast to *OsPT1*, the other three genes are increasingly expressed by low Pi stress and function at low Pi conditions. After being taken-up by rice roots, most Pi should be transported into shoots and then distributed into various organs. This process is complex and requires the cooperation of many Pi transporters. *OsPT2* and *OsPT8* are both highly expressed in stele cells of rice roots and leaves of shoots, and they play essential roles in Pi root-to-shoot translocation and Pi homeostasis in shoots [29,30]. Moreover, *OsPT8* also contributes to Pi translocation from the panicle axis to the rice hull. Also, *OsPT11* and *OsPT13* appear to be involved in *arbuscular mycorrhizal* fungal symbiosis [31]. Although studies of *Pht1* family genes in rice have lasted decades, there are still a few Pi transporters that need to be characterized. In this report, we investigated the function of *OsPht1;4* (accession no. AF 536964) in rice. We found that *OsPT4* is a functional Pi transporter localized mainly in the plasma membrane of exodermis cells in rice roots. Overexpressing *OsPT4* induced an increase in Pi concentration at different Pi levels. We concluded that *OsPT4* is involved in Pi uptake from an external solution.

## Material and Methods

### Plant Materials and Growth Condition

Based on the *Oryza sativa* ssp. *Japonica* cv. *Nipponbare* background, overexpression and knockdown lines of *OsPT4* were obtained via *Agrobacterium tumefaciens*-mediated transformation [32].

Standard rice culture solution was used in hydroponic experiments. The dispensation of culture solution was as follows: 1.44 mM NH_4_NO_3_; 0.5 mM K_2_SO_4_; 1.0 mM CaCl_2_; 1.6 mM MgSO_4_; 0.17 mM Na_2_SiO_3_; 0.3 mM NaH_2_PO_4;_ 50 μM Fe-EDTA; 0.06 μM (NH_4_)_6_Mo_7_O_24_; 15 μM H_3_BO_3_; 8 μM MnCl_2_; 0.12 μM CuSO_4_; 0.12 μM ZnSO_4_; 29 μM FeCl_3_; and 40.5 μM citric acid (pH 5.5)[33]. The transgenic lines were grown in solution containing different concentrations of phosphate with 0.3 mM NaH_2_PO_4_ (normal treatment), 1.5 mM NaH_2_PO_4_ (high-Pi treatment [HP]), and 0 mM NaH_2_PO_4_ (low-Pi treatment [LP]). The solution was renewed every 5 days.

### Microarray Expression Profile

To analyze the expression pattern of *OsPT4*, genome-wide expression data were searched in the CREP database (http://crep.ncpgr.cn/crep-cgi/home.pl) [34]. All these data were generated by hybridizing Affymetrix whole-genome arrays from RNA samples obtained from 27 tissues collected throughout the life cycle of three genotypes of cultivated rice: Minghui 63, Zhenshan 97, and their hybrid Shanyou 63.

### Histochemical Localization of GUS Expression

The construct of *OsPT4* promoter *GUS* was transformed into *Nipponbare* via *Agrobacterium tumefaciens—*mediated transformation [35]. The transgenic lines were grown in solution with or without Pi and the tissues and organs were incubated in X-Glu strain buffer at 37°C overnight [36]. To observe the cellular expression patterns, the stained tissues were rinsed in 70% ethanol for 24 hours, embedded in agarose gel, and then sectioned. The stained tissues were photographed using DFC290 stereomicroscope with a color CCD camera and sections were visualized with a digital camera DXM1200C.

### Subcellular Location of *OsPT4*

The coding sequence of *OsPT*4 (LOC_Os04g10750) from the RGAP database (http://crep.ncpgr.cn/crep-cgi/home.pl) was amplified and transformed into vector pM999-GFP. The preparation and transfection of rice protoplasts were performed as described [37]. Briefly, 0.1 mL of protoplast suspension (approximately 1×10^5^ cells) was transfected with 10 μg of DNA via 0.12 mL 40% PEG. After 12 to 24 hours of incubation at 22°C, the cells were observed by a confocal laser scanning microscope (TCS SP2).

### RNA Extraction and Real-Time PCR

Total RNA was extracted using TRizol regent (Invitrogen). According to the manufacturer’s instructions, 3 μg of total RNA was used to synthesize the first-strand cDNA in 20 μl of reaction mixture using M-MLV reverse-transcriptase (Invitrogen). Real-time PCR was performed using the SYBR Premix Ex Taq^TM^ (TaKaRa) with the gene-specific primers listed in Table 1. The real-time PCR reaction was performed on an Applied Biosystems 7500 PCR instrument. The rice Ubiquitin 5 gene was used as the internal control.

**Table 1:** Primers used in this study.

Primer Name	Sequence (5`-3`)
OE-*OsPT4*-F	ATGGATCCAATTGCAGCGAGATTTTGTC
OE-*OsPT4*-R	TACTGCAGTGGTACACTAGCAGAACCAGAA
GFP*- OsPT4*-F	TCTAGAATGGCCGGCGAGCTCAAGGTG
GFP*- OsPT4*-R	TCTAGAAGCTGGCGGCGCCGGC
pyes2*- OsPT4-*F	AAGCTTATTGCATATTGCAGAGTAGCTG
pyes2*- OsPT4-*R	GGATCCATGATTGACCATTTGGGTTTT
GUS*- OsPT4-*F	TGATTGATGAAACTGCTGCTG
GUS*- OsPT4-*R	ACATATCCAGCCATGCACACT
qRT*-OsPT4-*F	GCAACGTCATCGGGTTCTTCTTCA
qRT*-OsPT4-*R	ACATATCCAGCCATGCACACT
qRT*-OsPHO2-*F	GGCTATCGGAACTTATGG
qRT*-OsPHO2-*R	AAGAAGGCAGAGGAGGTATC
qRT*-OsPHR2-*F	CGCTTTGTAGATGCTGTCAATC
qRT*-OsPHR2-*R	AGACCCTCATCACATCCTCATTATC
qRT*-OsSPX1-*F	GACCAGCTTCTACCATCAAACG
qRT*-OsSPX1-*R	AGTTCCTGCTGCTCCTCTGG
qRT*-Ubiquitin-*F	AACCAGCTGAGGCCCAAGA
qRT*-Ubiquitin-*R	ACGATTGATTTAACCAGTCCATGA

Functional Complementation Assay of *OsPT4* in Yeast

The functional complementation experiment was performed to explore the Pi transport activity of *OsPT4*. The *OsPT4* open reading frame was first cloned into pGEM-T Easy vector and digested with xbaI, and then correctly introduced into vector *pYES2*. The resulting plasmid was transformed into yeast strain *Δpho84* (Mat a; his3*Δ*1; leu2*Δ*0; met15*Δ*0; ura3*Δ*0; YML123c::kanMX4) and its wild-type BY4741 (Mat a; his3*Δ*1; leu2*Δ*0; met15*Δ*0; ura3 *Δ*0) purchased from EUROSCARF (http://web.uni-frankfurt.de/fb15/mikro/euroscarf/index.html) [38]. The *pYES2-OsPT4* and control cells were grown to the logarithmic phase and then subjected to yeast nitrogen base liquid medium with different Pi concentrations of 0 μM and 75 μM, respectively. Bromcresol purple was used as the pH indicator in the medium.

### Measurement of Pi Concentration in Plants

The measurement of inorganic Pi was performed as described previously with some modifications. Fresh samples were milled in liquid nitrogen and were not placed on ice until they thawed. Then, the milled samples were homogenized in 10% (w/v) perchloric acid: 5% (w/v) perchloric acid (1:9) and placed on ice for 30 minutes. After centrifugation at 10,000*g* for 10 minutes at 4°C, the supernatant was used for Pi measurement using the molybdenum blue method. The working fluid is a 6:1 ratio intermixture of 0.4% (w/v) ammonium molybdate dissolved in 0.5 M H_2_SO_4_ mixed with 10% ascorbic acid. Two milliliters of intermixture was added to 1 mL of sample solution, incubated in a water bath at 42°C for 20 minutes, and then cooled on ice. Sample absorbance was measured at 820 nm, and Pi concentration was calculated by normalization to fresh weight values [39]. For the measurement of total Pi concentration in the rice, about 0.2 g dry samples were used following the method described by Chen et al [40].

## Results

### The Expression Pattern of *OsPT4* in Different Tissues of Rice

**Fig 1:**
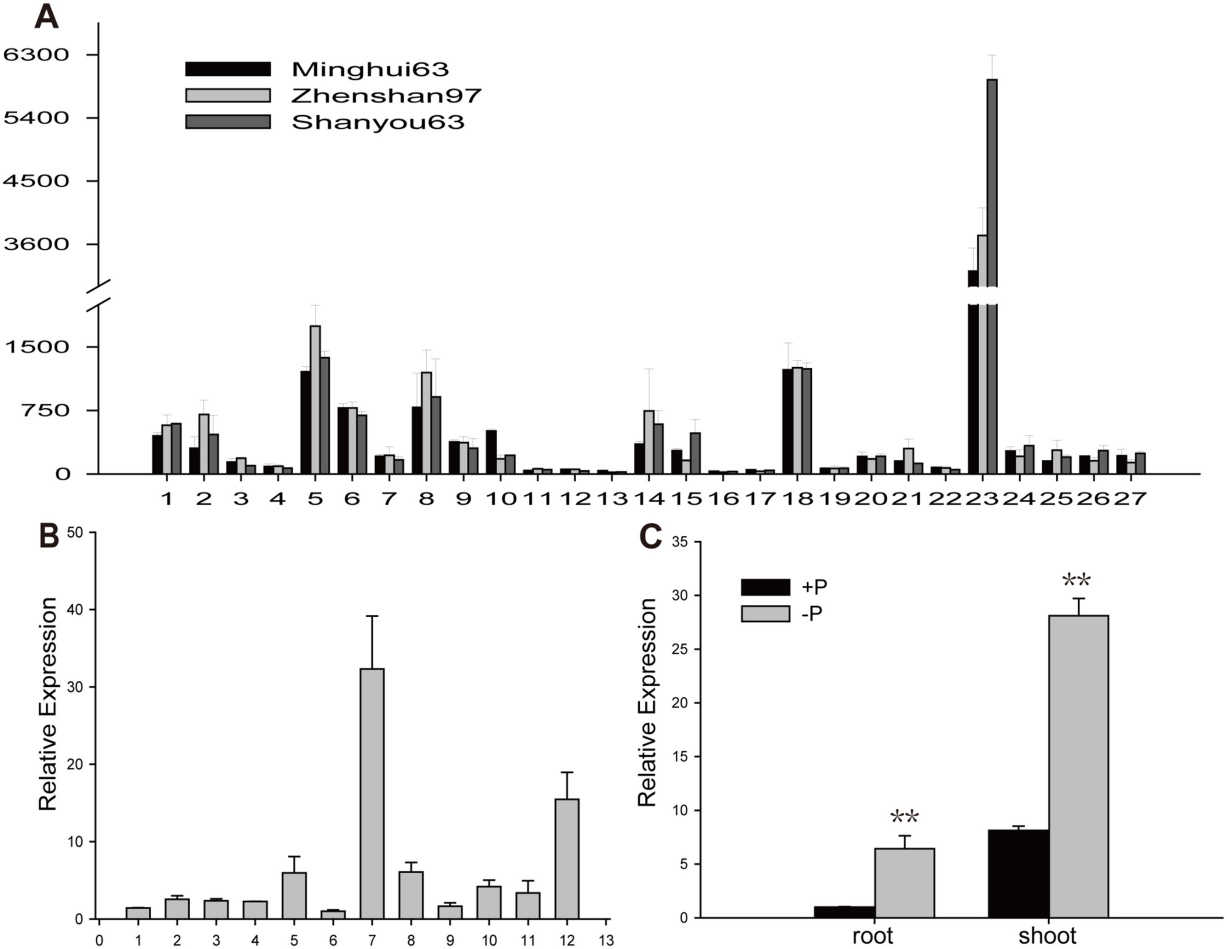
Expression profile of *OsPT4* and expression pattern in response to Pi availability. A: The Expression profile of *OsPT4* gene exhibited tissue- or organ-preferential expression patterns in 27 tissues covering the rice lifecycle based on average signal values in three rice cultivars: Minghui 63, Zhenshan 97 and Shanyou63, respectively. For relevance, there were 27 tissues analyzing in the study: (1) calli at 15 d after the subculture stage; (2) resistance calli at the screening stage; (3) calli at 5 d after the regeneration stage; (4) seed germination after 72 h of the stage; (5) embryo and radical after the germination stage; (6)leaf and root at three-leaf stage; (7) shoot at the seedling with two-tillers stage; (8) root at the seedling with two-tillers stage; (9) leaf at the 1-mm young panicle stage; (10) sheath at the 1-mm young panicle stage; (11) panicle at the 1-mm young panicle stage; (12) panicle at the pistil/stamen primordial differentiation stage; (13) panicle at the pollen/mother cell formation stage; (14) leaf at the 4–5-cm young panicle stage; (15) sheath at the 4–5-cm young panicle stage; (16) panicle at the 4–5-cm young panicle stage; (17) stem at 5 d before the heading stage; (18) flag leaf at 5 d before the heading stage; (19) stem at the heading stage; (20) panicle at the heading stage; (21) hull at 1 d before the flowering stage; (22) stamen at 1 d before the flowering stage; (23) flag leaf at 14 d after the heading stage; (24) spikelet at 3 d after the pollination stage; (25) endosperm at 7 d after the pollination stage; (26) endosperm at 14 d after the pollination stage; and (27) endosperm at 21 d after the pollination stage. B: The relative expression of *OsPT4* in different 12 tissues of *Nipponbare*. There were 12 tissues analyzing in the study: (1)flag leaf at 5 days before heading; (2)young panicle at 5 days before heading; (3)flag leaf at heading stage; (4)panicle at heading stage; (5)stem at heading stage; (6)panicle at one day before flowering; (7) flag leaf at 7 days after pollination; (8)culm at 7 days after pollination; (9) spikelet at 7 days after pollination; (10) culm at 14 days after pollination; (11)spikelet at 14 days after pollination; (12) root at 14 days after pollination. C: Detection of the expression alternation of *OsPT4* in wild-type plants roots and shoots by real-time PCR analysis. Plants were grown to five-leaf stage in full nutrient solution, then transplanted into the solution without Pi 21 days. Total RNA were extracted from plant tissues which were sampled at different time. Error bars indicate ±SD (n = 3). (**p<0.01).

To clarify *OsPT4* gene function in detail, the expression patterns in different tissues were extracted from the CREP database; the microarray data were verified by quantitative PCR to reveal the expression profile of *OsPT4*. The average signal values in Fig 1A were tested in 27 tissues or organs over the entire lifecycle of the rice plant in three genotypes: Minghui 63, Zhenshan 97, and Shanyou 63. Each sample was tested with two biological repeats. In general, *OsPT4* exhibited the strongest expression signals in vegetative organs, such as flag leaves and roots. *OsPT4* also showed a moderate expression level in calli, embryo, and radical. We also tested the transcript level of *OsPT4* in different tissues of *Nipponbare* by Real-time PCR. Consistent with prior results, the highest expression of *OsPT4* was observed in flag leaves, followed by roots (Fig 1B).

According to previous work, the expression of *Pht1* family genes is largely increased under phosphate-deficient conditions [8]. To investigate whether *OsPT4* is affected by low Pi treatment, we performed a time-course experiment under Pi-free and Pi-resupplied conditions. Real-time PCR showed that the transcript level of *OsPT4* in roots was not significantly changed when rice plants were hydroponically cultivated under Pi-free conditions for 7 days, but the expression of *OsPT4* in shoots increased with extended Pi deficiency, and quickly returned to normal levels after Pi was resupplied (S1 Fig). However, when the Pi-free conditions extend for 21 days, the transcript level of *OsPT4* increased significantly both in shoots and roots (Fig 1C).

**Fig 2:**
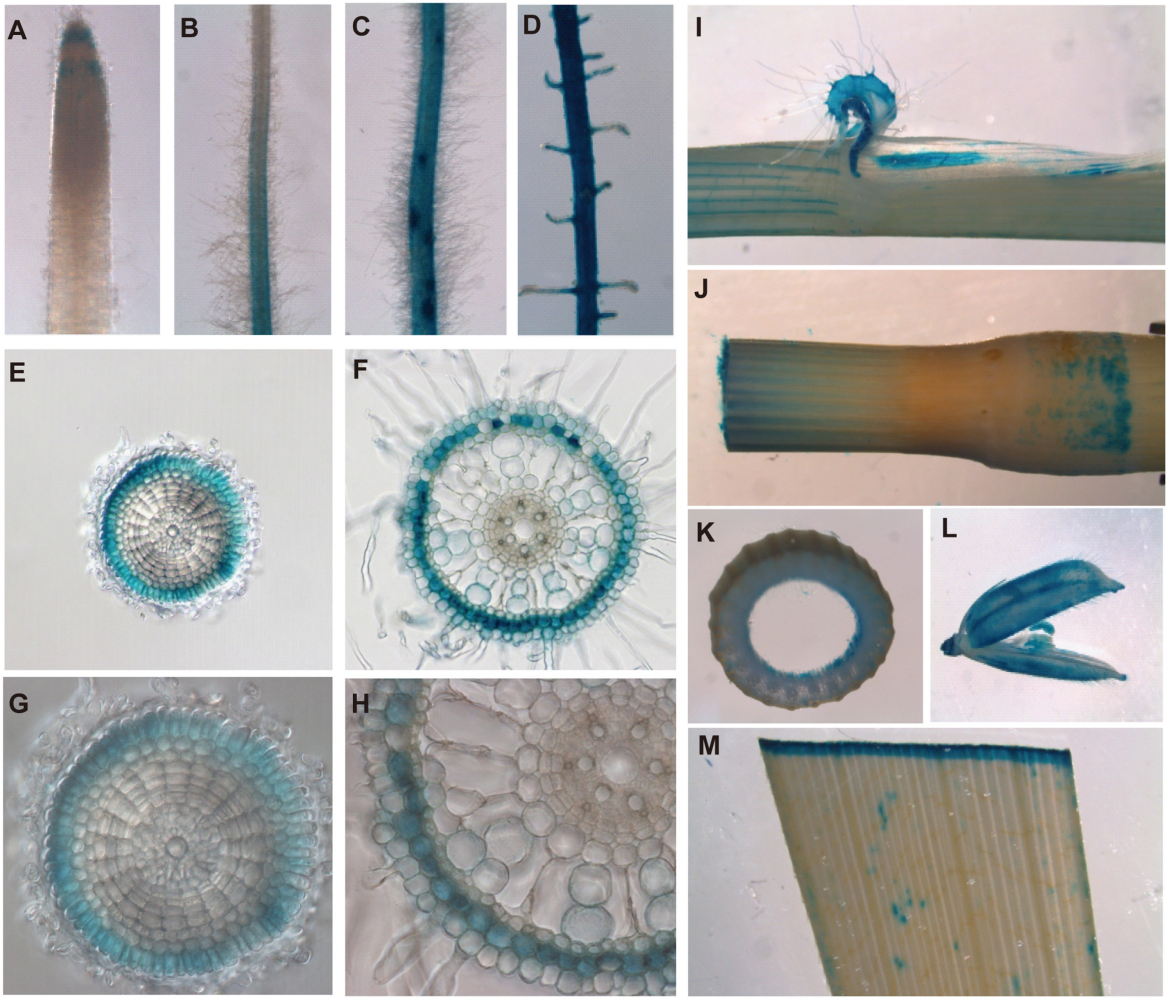
Tissue localization of *OsPT4*. GUS staining of transgenic plants harboring the *OsPT4* promoter. *GUS* fusion was observed. Expression of *OsPT4* was shown in different tissues of rice supplied with normal Pi for 21 days. A: Root tip. B: The junction between the meristematic and elongated zones. C: Branching of lateral root. D: Lateral root. E: Transverse section of root tip. F: Transverse section of root maturation zone. G and H: Enlarged images of (E) and (F). I: Ligule. J: Internodes of culm. K: Transverse section of culm. L: Caryopse and stamen. M: Leaf blade.

For histochenmical analysis, a construct containing a 1990-bp promoter and a GUS reporter gene was transformed into rice (cv *Nipponbare*) by *Agrobacterium*-mediated transformation. The transgenic plants carrying *OsPT4*::*GUS* were cultured in nutrient solution and stained for GUS activity. Strong GUS activity was detected in roots, leaves, ligules, stamens, and caryopses under sufficient Pi conditions (Fig 2), consistent with the expression profile described. In roots, *OsPT4* was mainly expressed in the tip and the mature zone of roots. We performed transverse sections of these two zones and found that *OsPT4* was specifically localized to the exodermis layer (Fig 2E and 2F). Since the transcript level of *OsPT4* was significantly increased under Pi-deprived condition, the GUS activity of the transgenic plants at Pi-free condition for 21 days was detected. Consistent with the result shown by Real-time PCR, the GUS activities in roots and shoots were both enhanced under Pi-deprived condition. Moreover, GUS activity was remarkably observed in root cortex of plants cultivated under Pi-deprived conditions whereas it was not detected in the root cortex under Pi- replete conditions (S2 Fig).

### Subcellular Location of OsPT4

**Fig 3:**
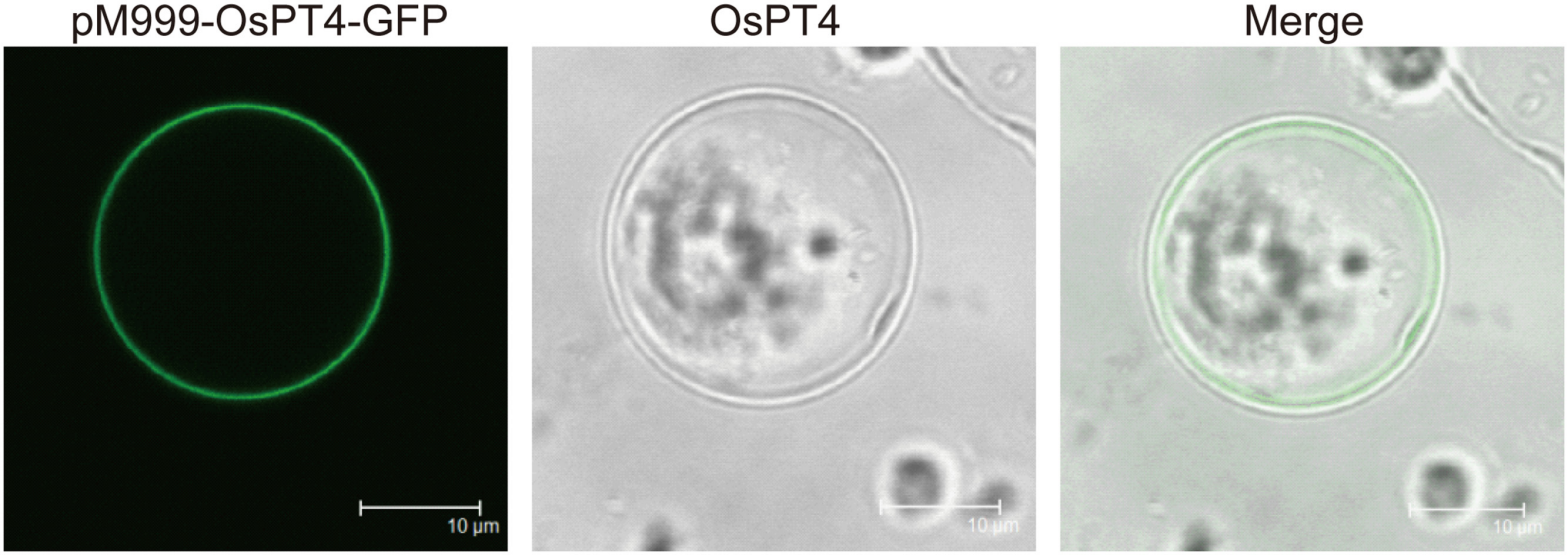
Subcellular localization of *OsPT4* protein. Subcellular localization of *OsPT4* protein was determined in rice protoplasts. The confocal image was acquired using a confocal laser scanning microscope (TCS SP2).

Plant Pht1 members were predicted to be located on the plasma membrane. For confirmation of the subcellular location of *OsPT4*, we created an *OsPT4*::*GFP* construct at C-terminal and transfected it into rice protoplasts. As expected, the green fluorescence of *OsPT4* was observed in the plasma membrane by laser scanning confocal microscopy (Fig 3).

### Functional Assay of *OsPT4* in Yeast

**Fig 4:**
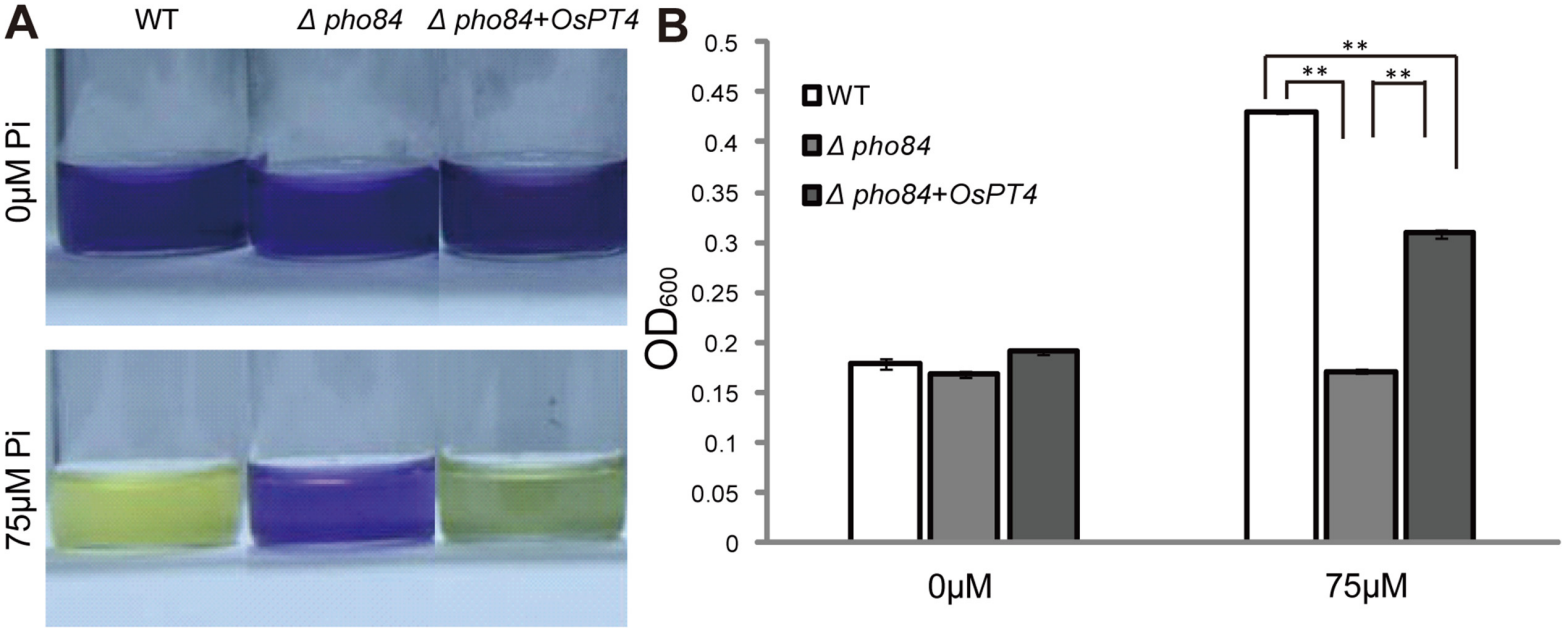
Function expression of *OsPT4* in yeast. The yeast cells were grown in solution that had been stained for acidification. The pH indicator, bromocresol purple, did not change from blue to yellow until the yeast cells had significant growth in culture. The medium contained 0 μM and 75 μM Pi, respectively. A: Color shifts in wild-type (WT), yeast strain *pho84* (control), and *pho84+OsPT4*, which express *OsPT4* in *pho84* grown on synthetic defined (SD) Ura mediums. B: Growth conditions of wild-type, *pho84*, and *pho84* transformed with pYES2-*OsPT4* generated in a 24-hour liquid culture under 0μM and 75μM Pi conditions. Statistically significant differences are indicated: **p<0.01, Student’s one-way ANOVA analysis.

Because *OsPT4* belongs to the *Pht1* family, which is always involved in Pi transport, a functional complementation analysis of the yeast mutant was performed. Yeast cell growth was well-correlated with acidification of the liquid growth media, as indicated by the color shift of the pH indicator bromocresol purple. Bromocresol purple changes its color from blue to yellow at pH from 6.3 to 5.8. We expressed the *OsPT4* in the yeast mutant strain *Δpho84*, which is defective in high-affinity Pi transport. The mutant yeast containing *OsPT4* and empty vector were grown in a yeast nitrogen base medium with various concentrations of Pi over 10 hours at pH 7.0 The pH indicator did not change from blue to yellow until the yeast cells had significant growth in culture. The colors of these three yeasts containing wild-type and *Δpho84* with and without *OsPT4* were all blue at the Pi concentration of 0 μM, revealing that little or no yeast cells had grown in medium without Pi. After increasing the Pi concentration of the medium to 75 μM, the color changed to yellow, with wild-type and *Δpho84* expressing *OsPT4* rather than mutant yeast, suggesting that the expression of *OsPT4* restored the yeast growth rate (Fig 4A). To further confirm this result, absorbance of these different yeasts at 600 nm was tested and showed that *OsPT4* could partially complement the function of yeast Pi transporter PHO84 (Fig 4B).

### Overexpression of *OsPT4* Increased Pi Accumulation in Roots

**Fig 5:**
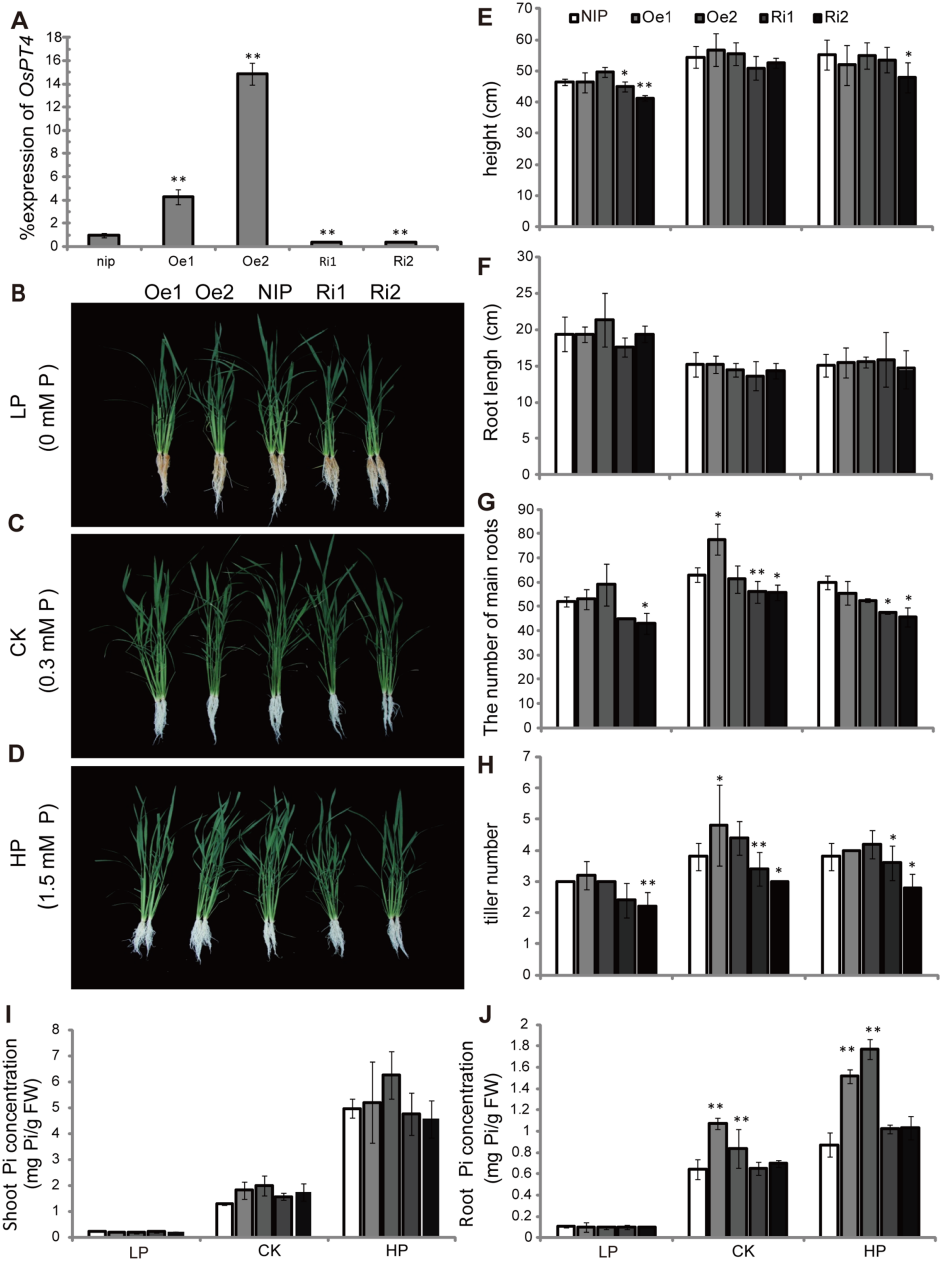
Characterization of wild-type and transgenic plants. A: Expression of OsPT4 in transgenic plants. Seven-day-old seedlings were transferred to nutrient solution for 2 weeks. RNA was extracted from the shoots of the seedling. Oe1, Oe2, Ri1, and Ri2 represented independent *OsPT4*-overexpressing and RNA interference lines. Relative expression levels are shown as percentages compared with wild-type shown as 100% expression. B, C, and D: Growth of wild-type and transgenic plants. Plants were grown in nutrient solutions to which 0 mM Pi (LP), 1.5 mM Pi (HP), and 0.3 mM Pi (CK) were added for 21 days. E–H: Phenotypic analysis of OsPT4 transgenic plants. Height, root length, number of main roots, and tiller numbers were obtained from the 21-day-old wild-type and transgenic plants grown in nutrient solutions with different Pi concentrations. Five plants per line were measured. I and J: The Pi concentration of shoots and roots in wild-type and transgenic plants. Data are means ± SD of five biological replicates. Values are significantly different from those of wild-type: *P<0.05 and **P<0.01. (one-way ANOVA).

To further examine the exact role that *OsPT4* plays in rice Pi uptake and translocation, we generated overexpression lines using the CaMV 35S promoter (*OsPT4*-Oe) and knockdown lines using RNAi (*OsPT4*-Ri) in *Nipponbare* via *Agrobacterium tumefaciens*-mediated transformation. Expression levels of the *OsPT4* gene in the transgenic lines were tested by real-time PCR, and two *OsPT4*-Oe lines (Oe1 and Oe2) and two *OsPT4*-Ri lines (Ri1and Ri2) were selected for further study. Under sufficient Pi conditions, the transcript levels of *OsPT4* in the two *OsPT4*-Oe lines were 4.1-times and 14-times higher than those of wild-type, respectively, whereas the transcript abundance of *OsPT4* decreased by 70% in *OsPT4*-Ri plants (Fig 5A).

The transgenic plants were initially grown for 1 week in complete culture and then shifted to culture with HP, LP and normal Pi for 21 days. Using different Pi treatments, the phenotypes of *OsPT4*-Oe plants, including plant height, root length, tiller number, and the number of main roots, showed no obvious difference compared with wild-type (Fig 5E–5H). No significant differences were found in plant height and root length between *OsPT4*-Ri lines and wild-type plants (Fig 5C). However, the number of main roots and tiller numbers were decreased in *OsPT4*-Ri lines (Fig 5G and 5H).

Pi concentrations were measured in shoots and roots of the 21-day-old transgenic plants grown with different Pi levels. When grown without Pi, all the lines possessed extremely low Pi concentrations both in roots and shoots and no significant difference in Pi concentrations were found among all the lines (Fig 5I and 5J). However, transgenic plants with overexpression of *OsPT4* showed a increased Pi accumulation in roots when grown in normal and HP conditions. Interestingly, higher Pi seemed to cause a more significant increase of Pi in *OsPT4*-Oe plants in comparison with wild-type: the Pi concentration of *OsPT4*-Oe lines increased 1.6-times and 1.3-times, respectively, with 0.3 mM Pi, and increased 1.73-times and 2.1-times with 1.5 mM Pi. However, no significant difference was found in shoots of transgenic and wild-type plants grown with these two Pi concentrations. Additionally, the Pi concentrations in *OsPT4*-Ri plants were similar to those of wild-type under different Pi conditions.

### Alteration of Pi Concentration in *OsPT4* Transgenic Plants Grown in Field

**Fig 6:**
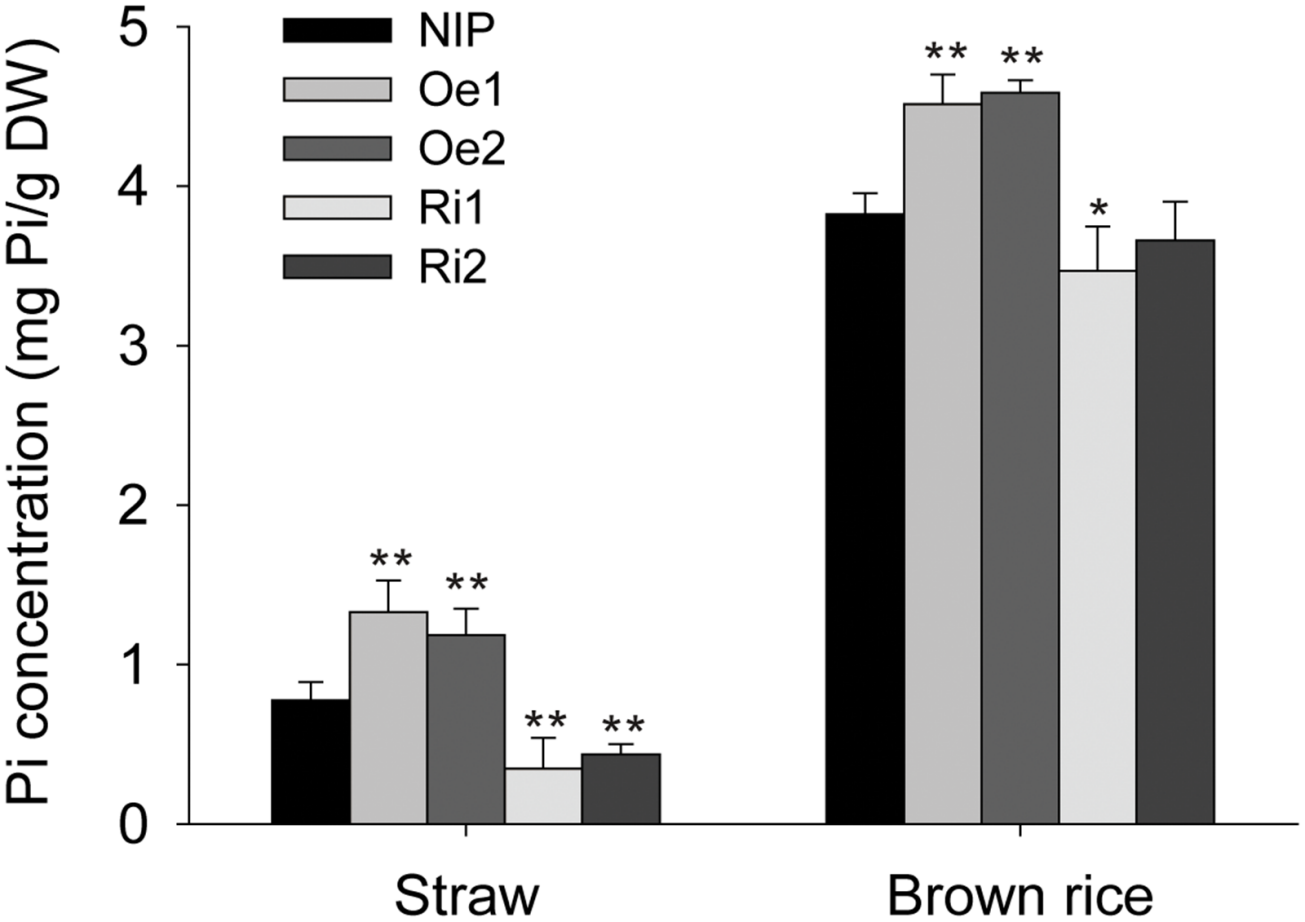
Pi concentration of the wild-type and transgenic plants in field. The rice including wild type and transgenic plants were grown in field. When plants grown to maturity, the phosphate concentration of straws and brown rice were measured. Data are means ± SD of five biological replicates. Values are significantly different from those of wild-type: *P<0.05 and **P<0.01. (one-way ANOVA).

The fact that the Pi concentration of *OsPT4*-Oe plants accumulated in roots suggested that *OsPT4* may play an important role in rice Pi uptake. To understand the function of *OsPT4* in Pi uptake, we measured the Pi concentration of wild type and *OsPT4* transgenic plants grown in field with normal P concentration (available Pi of 14mg/kg soil). The Pi concentration of transgenic plants in both straw and brown rice changed (Fig 6). The Pi concentration doubled in straw of the *OsPT4*-Oe plants compared with the wild type plants, whereas the straw Pi concentration in *OsPT4*-Ri decreased to about half of that in wild type plants. Furthermore, the Pi concentration of brown rice in *OsPT4*-Oe plants increased 26% compared with wild type plants, whereas the Pi concentrations in *OsPT4*-Ri plants were slightly decreased. The biomasses of wild type and OsPT4 transgenic plants had no significant difference (S3 Fig).

### Altered Expression of Pi-signaling Genes in *OsPT4* Transgenic Plants

**Fig 7:**
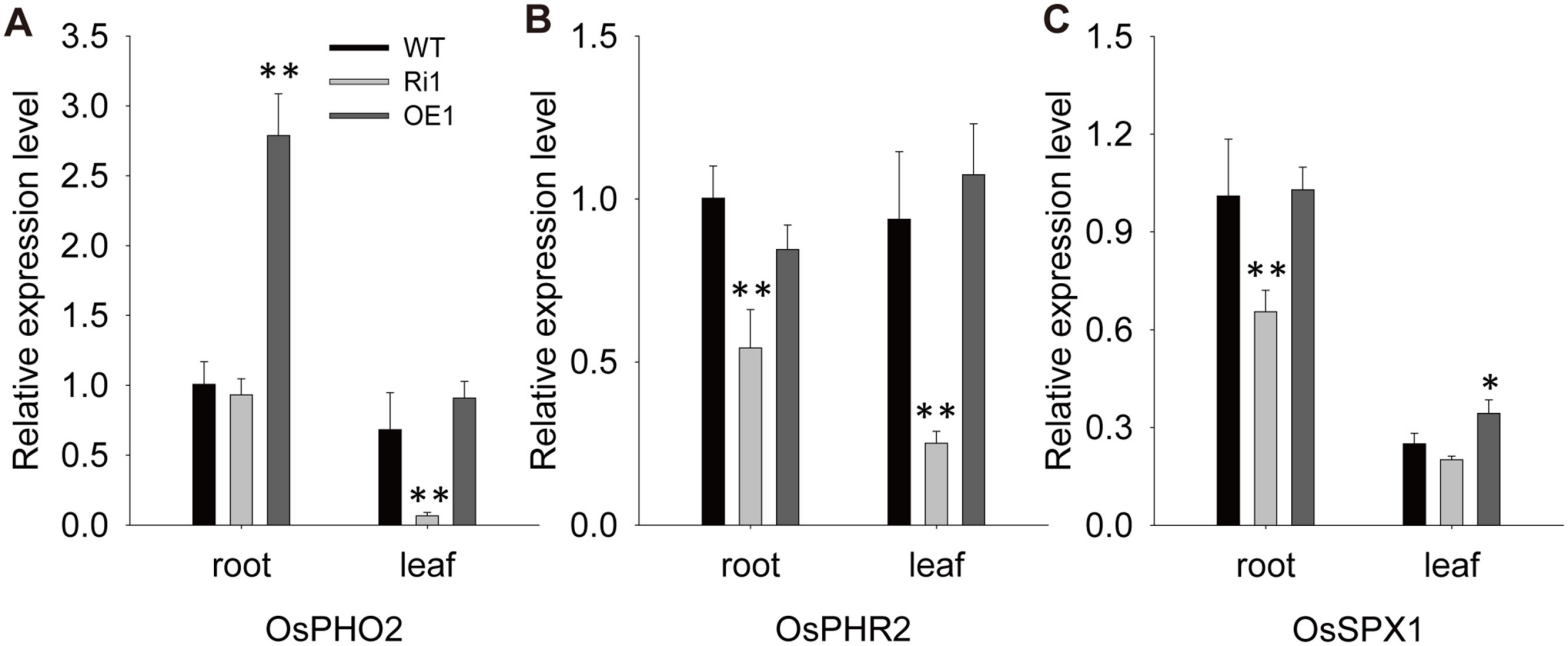
Expression of genes in wild-type transgenic plants under normal Pi condition. Expression of central transporters affecting Pi absorption and homeostasis in WT and transgenic plants. Error bars indicate ±SD (n = 3). (**p<0.01 and *P<0.05, one-way ANOVA).

It was revealed that many important central regulators affecting Pi absorption and homeostasis had been functionally characterized in the pathways for Pi signaling, such as *OsPHO2*, *OsPHR2*, and *OsSPX1*. Therefore, we tested the expression of these three genes in transgenic plants and found that their expressions were changed in some way. The transcript level of *OsPHO2* was increased in roots of *OsPT4*-Oe lines and dramatically decreased in leaves of *OsPT4*-Ri plants (Fig 7A). Additionally, *OsPHR2* showed down-regulated expression in *OsPT4*-Ri lines, and the expression of *OsSPX1* decreased slightly only in the roots of *OsPT4*-Ri plants (Fig 7B and 7C).

## Discussion

### *OsPT4* Is a Pi Transporter Involved in Pi Uptake

*Pht1* family genes are present in nearly every living organism and are well-known to be related to Pi transport. In rice plants, there are approximately 13 members belonging to the *Pht1* family, and these genes are expected to be well-organized with a clear division of labor in Pi uptake and translocation [9]. A few members of the rice *Pht1* family have shown the ability to transport Pi, and their exact roles in rice have also been clarified; *OsPT1*, *OsPT2*, *OsPT6*, and *OsPT8* are all able to transport Pi and function in Pi uptake and translocation [26,28,29]. However, the roles of many other members of the rice *Pht1* family are still unclear. In the present study we identified *OsPT4* (another rice *Pht1* family gene), which has a functional role in Pi transport. *OsPT4* was shown here to be a functional Pi transporter and to contribute to Pi absorption. This conclusion is based on the following findings: (1) *OsPT4* is an influx Pi transporter, at least in yeast, based on the fact that *OsPT4* can rescue yeast growth when expressed in yeast mutant *pho84* (Fig 4); (2) *OsPT4* encodes a plasma membrane protein, based on the GFP fluorescence from *OsPT4*-GFP fusion protein transformed into rice protoplasts (Fig 3); (3) *OsPT4* is highly expressed in roots and specific to exodermis cells, based on the GUS staining analysis (Fig 2); (4) overexpression of *OsPT4* in rice plants significantly increases root Pi concentration at different Pi levels compared with wild-type plants (Fig 5J) and (5) the Pi concentration of *OsPT4* transgenic plants strictly respond to the *OsPT4* transcripts change under field condition, overexpression of *OsPT4* result in higher phosphate accumulation and suppression of *OsPT4* decrease the total Pi amounts (Fig 6).

### Possible Role of *OsPT4* in Pi Translocation and Homeostasis

Pi is one of the most important elements for rice. In general, Pi is taken-up by rice roots, loaded into xylem, translocated to the shoots through root pressure and leaf transpiration, and, finally, distributed into various organs [1]. These processes always require multiple Pi transporters to collaborate with each other. During uptake, at least four Pi transporters other than *OsPT4*, including *OsPT1*, *OsPT6*, *OsPT9*, and *OsPT10*, have been shown to contribute to absorbing Pi at different Pi levels [27,28,41]. However, *OsPT4* was the only transporter that was specific to exodermis cells in rice roots under normal Pi conditions, thus revealing that the role of *OsPT4* is different from that of many other *Pht1* family genes (S1 and S2 Figs). Rice roots have two types of sclerenchymatous cells, one at the endodermis (known as Casparian strip) and another at the exodermis. The latter in rice always acts as the first apoplastic barrier to avoid outer substance influx; therefore, the exodermis layer plays a key role in managing radial uptake of various mineral elements from the external solution, including Pi [42]. Thus, we concluded that *OsPT4* is probably constitutively expressed in the exodermis layer of rice roots and plays an important role in Pi uptake at different Pi levels (Fig 2E and 2F). Interestingly, *OsPT4* was expressed in cells of root cortex under long period Pi deficiency. Since root cortex is involved in both apoplast and symplast pathways, the induced expression of *OsPT4* in cortex would probably enhance the symplastic transport of Pi and consequently help rice plants to obtain more Pi at low Pi condition.

*OsPT4* also shows high expression levels in various organs of rice shoots, such as leaves, ligules, nodes, internodes, and panicles, and its expression level in shoots is significantly induced by Pi-free conditions (Fig 2). This suggests that *OsPT4* is possibly involved in Pi transport and translocation in these organs. Moreover, it is worth noting that *OsPT4* showed the highest (even much higher than that in roots) expression level in flag leaves. Flag leaf is an essential tissue for the growth of rice panicles, and it plays a key role in remobilization of many mineral elements, from leaves to developing grains [43]. Until now, no Pi transporter had been shown to be localized in flag leaf and responsive to the remobilization of Pi from flag leaf to panicles. In our work, overexpressing *OsPT4* resulted in much higher Pi concentrations in brown rice (Fig 6), although this could be a cumulative effect of increased root uptake, increased root-to-shoot translocation and enhanced mobilization of Pi from other tissues to panicles. Thus, we deduced that *OsPT4* was also probably involved in Pi remobilization from flag leaf to panicles. However, the exact mechanism will require further study.

Thorough reviews have shown that several Pi transporter genes are regulated by a few central regulators, such as *OsPHR1* and *OsPHR2*, *OsPHO1* and *OsPHO2*, and four SPX genes [44–46]. However, the expression of these regulator genes can also be affected by feedback from transcriptional changes in PT genes. In *OsPT4* transgenic plants, we detected transcripts of *OsPHR2*, *OsPHO2*, and *OsSPX1* and found that the expression of all three genes was changed somewhat (Fig 7). In shoots of plants overexpressing *OsPT4*, *OsPHR2* and *OsPHO2* expression levels were greatly down-regulated, consistent with the conclusion that *OsPT4* is probably involved in shoot Pi translocation. In roots, *OsPHR2* and *OsSPX1* are down-regulated in *OsPT4*-RNAi plants, whereas the expression of *OsPHR2* is up-regulated in *OsPT4*-overexpressed plants. The changes in expression levels of these regulator genes might be a possible reason why many other Pi transporter genes are transcriptionally affected in *OsPT4* transgenic plants.

## Supporting Information

S1 Fig Expression pattern in response to Pi availability. Detection of the expression alternation of *OsPT4* in wild-type plants roots and shoots by real-time PCR analysis. Plants were grown to five-leaf stage in full nutrient solution, then transplanted into the solution without Pi one week and re-supplied Pi one day. Total RNA were extracted from plant tissues which were sampled at different time. Error bars indicate ±SD (n = 3). (**p<0.01).
(TIF)

S2 Fig Tissue localization of *OsPT4* in different Pi conditions. GUS staining of transgenic plants harboring the *OsPT4* promoter. *GUS* fusion was observed. Expression of *OsPT4* was shown in different tissues of rice supplied with Pi (A-C) and without Pi (D-F) for 21 days. A and D: leaf blade. B and E: Transverse section of root maturation zone. C and F: Enlarged images of (H) and (K).
(TIF)

S3 Fig Biomass of WT and *OsPT4* transgenic plants in the field. The rice including wild type and transgenic plants were grown in field. When plants grown to maturity, the biomass of wild type and *OsPT4* transgenic plants were measured. Data are means ± SD of five biological replicates. Values are significantly different from those of wild-type: *P<0.05 and **P<0.01. (one-way ANOVA).
(TIF)
