# Supplementary figures and images for: The Phosphate Transporter Gene OsPht1;4 Is Involved in Phosphate Homeostasis in Rice
Source: PLoS One. 2015 May 13;10(5):e0126186. doi: 10.1371/journal.pone.0126186 (PMC4430236; doi:10.1371/journal.pone.0126186)

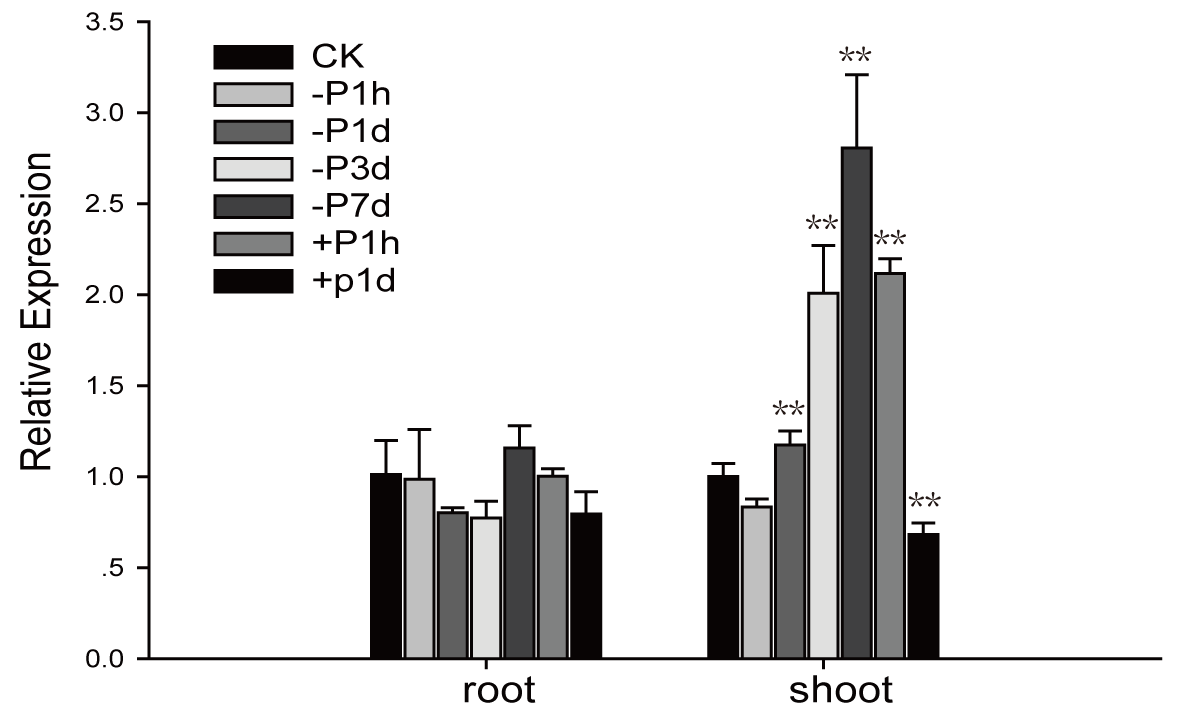

Supplement: S1 Fig — Detection of the expression alternation of OsPT4 in wild-type plants roots and shoots by real-time PCR analysis. Plants were grown to five-leaf stage in full nutrient solution, then transplanted into the solution without Pi one week and re-supplied Pi one day. Total RNA were extracted from plant tissues which were sampled at different time. Error bars indicate ±SD (n = 3). (**p<0.01). (TIF) [file pone.0126186.s001.tif]

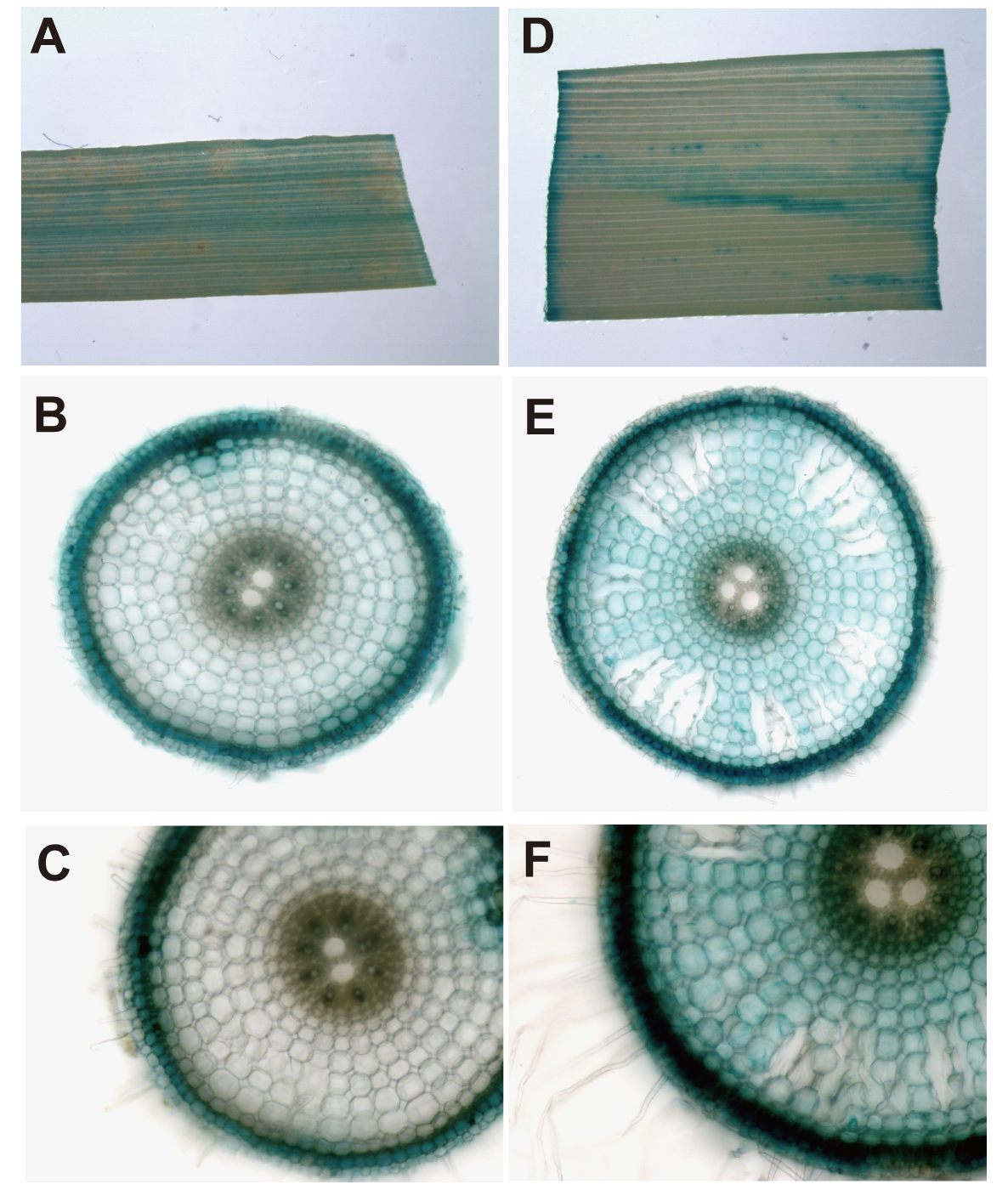

Supplement: S2 Fig — GUS staining of transgenic plants harboring the OsPT4 promoter. GUS fusion was observed. Expression of OsPT4 was shown in different tissues of rice supplied with Pi (A-C) and without Pi (D-F) for 21 days. A and D: leaf blade. B and E: Transverse section of root maturation zone. C and F: Enlarged images of (H) and (K). (TIF) [file pone.0126186.s002.tif]

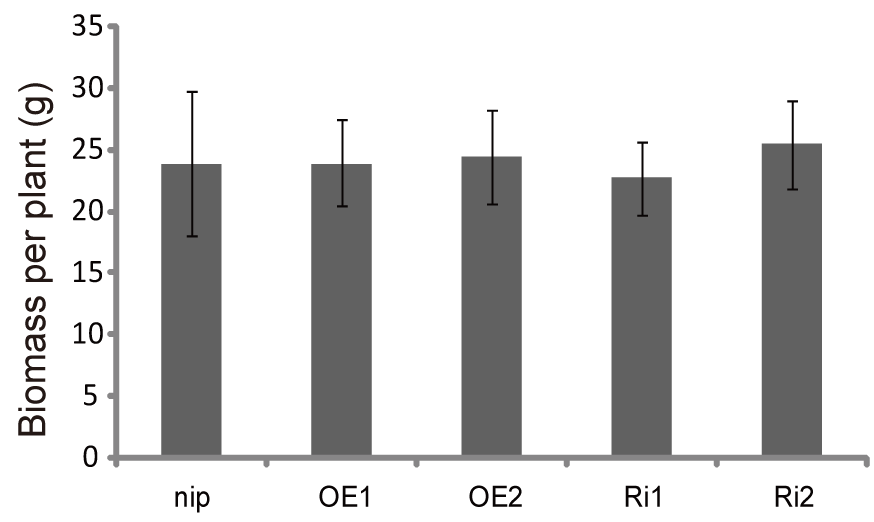

Supplement: S3 Fig — The rice including wild type and transgenic plants were grown in field. When plants grown to maturity, the biomass of wild type and OsPT4 transgenic plants were measured. Data are means ± SD of five biological replicates. Values are significantly different from those of wild-type: *P<0.05 and **P<0.01. (one-way ANOVA). (TIF) [file pone.0126186.s003.tif]
